# KILing in the name of embryonic growth: KIL transcription factors drive cell death in the maize endosperm

**DOI:** 10.1093/plcell/koaf133

**Published:** 2025-05-21

**Authors:** Sonhita Chakraborty

**Affiliations:** Assistant Features Editor, The Plant Cell, American Society of Plant Biologists

Life's journey within a single kernel of maize (*Zea mays*) is one of growth and sacrifice. As the developing embryo derives nutrition from the neighboring nutrient-rich starchy endosperm (SE), it expands further into the endosperm and occupies more space within the kernel. The cell layer bordering the endosperm and embryo—dubbed the endosperm adjacent to scutellum (EAS)—supplies the embryo with nutrients from the endosperm (Doll et al. 2020). After serving its purpose, the EAS accommodates the expanding embryo by regularly undergoing developmental programmed cell death (dPCD) (Figure A). Unlike Arabidopsis, where dPCD is followed by the complete elimination of cellular debris, maize EAS elimination is accompanied by the accumulation of cell wall components and remaining cytosolic components. In parallel, an additional round of cell death occurs in the SE, which is thought to be important for nutrient storage that will be useful later during seed germination (Doll and Nowack 2024). The distinct mechanisms and implications of these sequential cell death events in the maize endosperm during embryonic development are a big mystery. In their new work, **Nicolas M. Doll and colleagues (Doll et al. 2025)** explore the genetic regulation of EAS elimination and SE cell death in the maize kernel.

**Figure.**
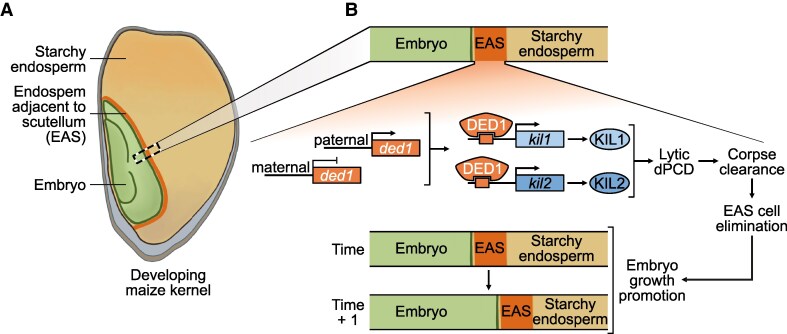
An overview of cell death events in the developing maize endosperm. **A)** Structure of developing maize kernel. **B)** DED1 and its targets, KIL1 and KIL2, regulate EAS dPCD and elimination during embryo expansion. Adapted from Doll et al. (2025), Figure 6.

The authors began their investigations by comparing the progression of cell death events at the SE and EAS. Before cell death is initiated, the protein bodies and starch granules are packed at the SE. Transmission electron microscopy images show that following SE cell death, the plasma membrane and organelles like the endoplasmic reticulum and mitochondria are not easily discernible, but protein bodies and starch granules persist at the cell corpses. Using a cell viability marker specifically expressed in the endosperm (*pronac130:mCherry-NLS)*, the authors traced a wave of cell death radiating outwards from the inner SE cells. In contrast to SE cell death, where structures are conserved, the dead EAS cells are completely eliminated. The authors explored the genetic regulation underlying the 2 forms of cell death by performing single-nucleus RNA-seq on maize endosperm. Of the dPCD-promoting transcription factors, *kil1* and *kil2* were strongly expressed at the EAS along with genes involved in regulating cell death processes (Doll et al. 2025). Indeed, this finding is in line with *kil*'s known function in cell death in maize, including during silk senescence (Šimášková et al. 2022).

The restricted expression of *kil1* and *kil2* at the EAS but not in the SE was intriguing and prompted the authors to investigate the role of KIL transcription factors during cell death in the endosperm. To this end, transgenic lines were generated that expressed a dominant-negative version of KIL1 named *kil1-SRDX*, wherein a transcriptional silencing motif, SUPERMAN REPRESSION DOMAIN X (SRDX), was fused to the C-terminus of KIL1. When *kil1-SRDX* was expressed in both EAS and SE, kernel phenotype was reminiscent of the *kil* loss-of-function phenotypes: smaller embryos and larger endosperms as less of the EAS was eliminated (Doll et al. 2025). Interestingly, the finding that SE death in *kil1-SRDX* lines is indistinguishable from wild-type kernels indicated that KIL regulates cell death at EAS but not the SE. Finally, to gain insights into the mechanistic basis of KIL-mediated EAS elimination, the authors turned their attention to the DOSAGE-EFFECT DEFECTIVE1 (DED1/ZmMYB73) transcription factor. The paternally derived DED1 is not only expressed in the EAS, but it has also been shown to enhance the expression of sugar transporters and to bind to the *kil1* and *kil2* promoters (Dai et al. 2022). Transiently expressing DED1 in maize protoplasts caused activation of the *kil1* and *kil2* promoters. Collectively, these data suggest that paternally expressed DED1-mediated activation of *KIL1* and *KIL2* enhances the elimination of the EAS layers from the endosperm during embryonic growth (Figure B).

The work of Doll et al. (2025) reveals a level of complexity in maize cell death that extends beyond what is known from Arabidopsis, which has a nonpersistent endosperm, with distinct genetic and regulatory controls, including potential parental influence. Further dissecting and manipulating these regulatory pathways, particularly at the EAS, is at the heart of finetuning optimal embryo growth and unlocking new strategies to improve agronomic traits critical for crop yield and seed quality (Nagasawa et al. 2013).

## Recent related articles in *The Plant Cell*:

- Bosch and Franklin-Tong (2024) explored the regulation of PCD in plant cells, with special emphasis on the roles of intracellular acidification and calcium signaling in this process.
- Wu et al. (2024) investigated how the maize transcription factors NAKED ENDOSPERM1 (NKD1), NKD2, and OPAQUE2 (O2) interact to regulate gene networks during endosperm development.
- Cheng et al. (2024) demonstrate that the maternally derived FERTILIZATION-INDEPENDENT ENDOSPERM1 (OsFIE1) modulates aleurone development and thickness in rice by depositing H3K27me3 marks on gibberellin biosynthesis genes.

## Data Availability

There are no new data associated with this article.

## References

[koaf133-B1] Bosch M, Franklin-Tong V. Regulating programmed cell death in plant cells: intracellular acidification plays a pivotal role together with calcium signaling. Plant Cell. 2024:36(11):4692–4702. 10.1093/plcell/koae245 39197046 PMC11530775

[koaf133-B20] Cheng X, Zhang S, E Z, Yang Z, Cao S, Zhang R, Niu B, Li QF, Zhou Y, Huang X–Y, et al Maternally expressed FERTILIZATION–INDEPENDENT ENDOSPERM1 regulates seed dormancy and aleurone development in rice. Plant Cell. 2024:37(1):koae304. 10.1093/plcell/koae304 39549266 PMC11663568

[koaf133-B2] Dai D, Mudunkothge JS, Galli M, Char SN, Davenport R, Zhou X, Gustin JL, Spielbauer G, Zhang J, Barbazuk WB, et al Paternal imprinting of dosage-effect defective1 contributes to seed weight xenia in maize. Nat Commun. 2022:13(1):5366. 10.1038/s41467-022-33055-9 36100609 PMC9470594

[koaf133-B3] Doll NM, Fierlej Y, Eekhout T, Elias L, Bellot C, Sun G, Grones C, Aesaert S, Coussens G, De Rycke R, et al KIL transcription factors promote endosperm elimination by lytic cell death to facilitate embryo growth in maize. Plant Cell. 2025. 10.1093/plcell/koaf162 40579219

[koaf133-B4] Doll NM, Just J, Brunaud V, Caïus J, Grimault A, Depège-Fargeix N, Esteban E, Pasha A, Provart NJ, Ingram GC, et al Transcriptomics at maize embryo/endosperm interfaces identifies a transcriptionally distinct endosperm subdomain adjacent to the embryo scutellum. Plant Cell. 2020:32(4):833–852. 10.1105/tpc.19.00756 32086366 PMC7145466

[koaf133-B50] Doll NM, Nowack MK. Endosperm cell death: roles and regulation in angiosperms. J Exp Bot. 2024:75(14):4346–4359. 10.1093/jxb/erae052 38364847 PMC7616292

[koaf133-B5] Nagasawa N, Hibara K, Heppard EP, Vander Velden KA, Luck S, Beatty M, Nagato Y, Sakai H. GIANT EMBRYO encodes CYP78A13, required for proper size balance between embryo and endosperm in rice. Plant J. 2013:75(4):592–605. 10.1111/tpj.12223 23621326

[koaf133-B6] Šimášková M, Daneva A, Doll N, Schilling N, Cubría-Radío M, Zhou L, De Winter F, Aesaert S, De Rycke R, Pauwels L, et al KIL1 terminates fertility in maize by controlling silk senescence. Plant Cell. 2022:34(8):2852–2870. 10.1093/plcell/koac151 35608197 PMC9338811

[koaf133-B7] Wu H, Galli M, Spears CJ, Zhan J, Liu P, Yadegari R, Dannenhoffer JM, Gallavotti A, Becraft PW. NAKED ENDOSPERM1, NAKED ENDOSPERM2, and OPAQUE2 interact to regulate gene networks in maize endosperm development. Plant Cell. 2024:36(1):19–39. 10.1093/plcell/koad247 PMC10734603 37795691

